# Oncogenic *Hras^G12V^* expression plus knockdown of *Cdkn2a* using ecotropic lentiviral vectors induces high-grade endometrial stromal sarcoma

**DOI:** 10.1371/journal.pone.0186102

**Published:** 2017-10-05

**Authors:** Laura P. Brandt, Joachim Albers, Tomas Hejhal, Antonella Catalano, Peter J. Wild, Ian J. Frew

**Affiliations:** 1 Institute of Physiology, University of Zurich, Zurich, Switzerland; 2 Zurich Center for Integrative Human Physiology, University of Zurich, Zurich, Switzerland; 3 BIOSS Centre for Biological Signalling Studies, University of Freiburg, Freiburg, Germany; 4 Clinic of Internal Medicine I, Hematology, Oncology and Stem Cell Transplantation, Faculty of Medicine, Medical Center - University of Freiburg, Freiburg, Germany; 5 Institute of Pathology and Molecular Pathology, University Hospital Zurich, Zurich, Switzerland; Universite du Quebec a Trois-Rivieres, CANADA

## Abstract

The uterine corpus represents the most common site for tumour development in the female genital system. Uterine neoplasms are categorised as epithelial, mesenchymal, mixed epithelial-mesenchymal or trophoblastic tumours. In this study we employed a mouse genetic approach using the MuLE lentiviral gene regulatory system to functionally test the ability of ecotropic lentiviruses to model epithelial and mesenchymal uterine malignancies *ex vivo* and *in vivo*. We discovered that MuLE lentiviruses efficiently infect uterine stromal cells but not endometrial epithelial cells when injected into the uterus of cycling, pseudopregnant or ovarectomized mice. Consistent with this cellular infection spectrum, we show that intra-uterine injection of ecotropic MuLE viruses expressing oncogenic *Hras*^*G12V*^ together with knockdown of *Cdkn2a* induce high-grade endometrial stromal sarcomas. These findings establish this approach as an efficient method of generating autochthonous mouse models of uterine sarcomas and in general for performing genetic manipulations of uterine stromal cells *in vivo*.

## Introduction

Early-stage uterine tumours are associated with an excellent prognosis after surgical treatment. However, for patients with advanced, metastatic or recurrent uterine cancer, limited therapeutic options are available [1,2]. To improve understanding of these diverse diseases it would be desirable to have a series of representative mouse models that reflect not only the different histological types of uterine tumours but also the underlying molecular genetics of each of these different tumour types. In recent years there has been some progress towards this goal.

The most prevalent tumours of the female reproductive system are carcinomas of the endometrium, which are classified in a dualistic model based on histological appearance and molecular differences [1]. Type I endometrioid tumours account for 80% of endometrial malignancies and are characterised by a relatively high survival rate, due partly to their frequent early detection following abnormal uterine bleeding. Several studies report that *PTEN* mutations occur early in the development of type I tumours [3–5]. In terms of generating rapid, flexible and accurate mouse models of this disease, it has been shown that the injection of adenoviruses expressing Cre into the uterine lumen of adult *Pten*^fl/fl^ mice caused the development of uterine carcinomas [6]. Tumour onset and severity can be accelerated by combining *Pten* deletion with *Kras* activation [7]. This combination of germline-based generation of Cre-regulatable genetic alleles plus viral-based delivery of Cre to somatic endometrial cells represents one approach to speed up the generation of mouse models of different molecular forms of endometrial carcinoma. Type II endometrial tumours are more common in older, non-obese women and, despite their rarity, have a worse outcome due to their highly invasive and metastatic nature. They comprise three different subtypes: serous, clear cell and undifferentiated carcinomas, each representing approximately 5–10% of all endometrial carcinoma cases [1]. Type II tumours are associated with both overlapping and distinct sets of genetic alterations to those found in type I tumours. They exhibit extensive somatic copy number alterations. In contrast to type I tumours, type II tumours are frequently characterised by early mutations in *TP53* [8–10]. The deletion of the *Trp53* gene in the mouse endometrium results in the formation of all histological subtypes of type II disease after 14–16 months [11].

Uterine mesenchymal tumours account for 3% of uterine tumours and are derived from the soft tissue of the uterine corpus, which comprises endometrial stroma, smooth muscle and blood vessels [12]. Uterine sarcomas are broadly classified as leiomyosarcomas, accounting for 60% of cases, endometrial stromal sarcomas, and undifferentiated sarcomas. These categories are defined by the presence of specific molecular alterations as well as by tumour morphology and prognosis [13]. Leiomyosarcomas are composed of cells which demonstrate smooth muscle differentiation. The majority of uterine leiomyosarcomas occur sporadically. Commonly found alterations include *MED12*, *HMGA2* and *PTEN* [14–16]. Patients with germline mutations in fumarate hydratase (*FH*) have an increased risk of developing uterine leiomyosarcomas as well as uterine leiomyomas [17].

Endometrial stromal sarcomas are composed of cells resembling the endometrial stroma and are far less frequent than smooth muscle tumours, such as leiomyosarcomas. They are characterized by chromosomal translocations, most commonly involving the zinc finger genes *JAZF1* and *SUZ1* [18,19]. Undifferentiated uterine sarcomas represent a very rare and aggressive category of uterine tumours. Considering their low incidence rate, there is little to no information about molecular alterations found in these tumours. They are diagnosed by exclusion after elimination of other high-grade uterine tumours with a sarcomatous component [20,21]. However, again using the approach of combined germline genetics plus viral delivery of Cre, injection of an adenovirus expressing Cre-recombinase into the uterus of mice harbouring conditional alleles of oncogenic *Kras* and *Trp53* caused the development of undifferentiated pleomorphic sarcoma [22,23].

In order to accelerate the process of generating genetically-complex autochthonous mouse models of human tumours we recently developed the MuLE lentiviral gene regulatory system, that facilitates combinatorial somatic genetics in cultured cells and *in vivo* in mouse tissues. As a proof of principle, we showed that this system could be employed to generate new mouse models of muscle-derived undifferentiated sarcomas that harbour alterations in multiple signalling pathways [24]. In the current study we investigated whether the MuLE system could also be applied to the setting of modelling of different forms of uterine tumours. We show that the MuLE system can be used to model endometrial sarcomas but does not appear to be easily applicable to modelling of uterine carcinomas.

## Materials and methods

### Mice

SCID/beige mutant mice (C.B-17/CrHsd-Prkdc^Scid^Lyst^bg-J^) and C57BL/6JRccHsd mice were obtained from Envigo. B6.Cg-Gt(ROSA)Sor^tm14(CAG-tdTomato)Hze^/J mice were obtained from the Jackson Laboratory. Vasectomized RjOrl:SWISS male mice were obtained from Janvier labs. Pax8-rtTA; LC1 mice [25], were obtained from Prof. Carsten Wagner, University of Zurich. For doxyclycline experiments, 6–8 week-old offspring of intercrosses between Pax8-rtTA; LC1 and B6.Cg-Gt(ROSA)Sor^tm14(CAG-tdTomato)Hze^/J mice were exposed to doxycycline (0.5 mg/ml) in drinking water supplemented with 5% sucrose for 5 days. Afterwards animals were kept on normal drinking water for another week. Mouse experiments were approved by the Veterinary Office of the Canton of Zurich under the licence 137/2013.

### Ovarectomy

Adult, cycling female B6.Cg-Gt(ROSA)Sor^tm14(CAG-tdTomato)Hze^/J mice were anaesthetized with isoflurane and the ovaries were exposed. Both uterine horns were ligated and the ovaries were removed. 2 weeks following surgery lentiviruses were injected into the uterus.

### Intrauterine viral injections

Approximately 4 week-old female mice were anaesthetized using isoflurane and the uteri were exposed by making a small incision. 10 μL of concentrated lenti- and adeno-viruses expressing different genetic manipulations were injected into one uterine horn with a 30G insulin syringe. The animals were euthanised 1–6 months after injection depending on the experiment. The uteri were harvested, fixed in 10% formalin, paraffin-embedded and cut in 5 μm thick sections. For the identification of the Cre-infected cells, B6.Cg-Gt(ROSA)Sor^tm14(CAG-tdTomato)Hze^/J uteri were fixed in 4% paraformaldehyde for 4–6 h, transferred to 30% sucrose overnight, embedded in OCT and cut in 5 μm thick sections.

### Allograft studies

For allograft experiments, 8 week-old female SCID/beige mice were anaesthetized by the inhalation of 2.5% isoflurane and 1x10^6^ cells resuspended in 50% Matrigel (BD, no. 354230, Eysins, Switzerland) were injected subcutaneously in a total volume of 150 μL. *In vivo* imaging as well as a caliper were used to follow tumour development over time.

### *In vivo* imaging studies

Noninvasive *in vivo* fluorescence and bioluminescence imaging was performed using the IVIS Spectrum (Perkin Elmer, Waltham, MA) together with the Living Image software (version 4.4). Mice were anaesthetized by using 2.5% isoflurane. During imaging, the isoflurane levels were reduced to 1.5%. All fluorescence measurements were performed in epi-fluorescence mode. For bioluminescence imaging, mice were injected subcutaneously with 150 mg/kg D-luciferin (Caliper, no. 122796, Waltham, MA) and imaged 15 min after injection. For the quantification of the total radiant efficiency, a region of interest was drawn around the mouse and the total radiant efficiency was automatically detected.

### Isolation and maintenance of pEEC

We used previously published protocols [26,27] with minor modifications for the isolation of primary endometrial epithelial cells from mice. Five to ten 3-week-old C57BL/6 mice were sacrificed by cervical dislocation and uterine horns were taken out, dissected in Hanks Balanced Salt Solution (HBSS, ThermoScientific, Waltham, MA) and cut open lengthwise. The fragments were then pooled and incubated with 0.5% Trypsin (Sigma Aldrich, St. Louis, MO)/ 2.5% pancreatin (Thermo Fisher Scientific, Waltham, MA) in HBSS for 60 min at 4°C and 60 min at 22°C. Digested uteri were transferred to ice-cold HBSS and vortexed for 30 sec to release epithelial cells. Uterine tissues were filtered through a 40 μm nylon mesh and epithelial cells were collected and centrifuged at 1.000 *g* for 5 min. Cells were then resuspended in basal medium containing DMEM/F12 (Sigma Aldrich, St Louis, MO) supplemented with 1 mmol/l HEPES (Thermo Fisher Scientific, Waltham, MA) and 1% penicillin/streptomycin (Sigma Aldrich, St Louis, MO). Cells were mechanically disrupted by pipetting until clumps of cells are observed under the microscope. Cells were then seeded in basal medium supplemented with 5 ng/ml EGF (PeproTech, Hamburg, Germany), 1 mg/ml insulin (Sigma Aldrich, St Louis, MO), 0.55 mg/ml transferrin (Sigma Aldrich, St Louis, MO), 0.5 μg/ml sodium selenite (Sigma Aldrich, St Louis, MO) (BIE—basal medium, ITS supplement and EGF) and 0.5% dextran-coated charcoal-stripped FCS (Biochrom AG, Berlin, Germany) and plated into cultures dishes. Cells were cultured in BIE plus 0.5% stripped FCS in an incubator at 37°C with saturating humidity and 5% O_2_.

### Immunohistochemistry

Immunohistochemical analysis was performed on formalin-fixed paraffin sections after antigen retrieval (5 min at 110°C in 0.1 M citrate buffer pH 6) and immunofluorescence on cells (IF-IC) and frozen sections (IF-F) on paraformaldehyde-treated cells or sections. The antibodies used in this study were anti-CD10 antibody (1:2000, PA5-47075, Thermo Fisher Scientific, Waltham, MA), anti-CD31 antibody (1:200, ab28364, abcam, Cambridge, UK), anti-CYCLIN D1 antibody (1:40, SP4, Thermo Fisher Scientific, Waltham, MA), anti-DESMIN antibody (1:100, D1033, Sigma-Aldrich, Darmstadt, Germany), anti-MYOD1 antibody (1:100, M3512, Dako, Santa Clara), anti-MYOGENIN antibody (1:500, M3559, Dako, Santa Clara), anti-alpha-SMOOTH MUSCLE ACTIN antibody (1:5000, ab5694, abcam, Cambridge, UK), anti-H-RAS antibody (1:100, GTX116041, GeneTex, Irvine, CA) and anti-VIMENTIN antibody (1:500, D21H3, Cell Signaling, Danvers, MA).

## Results

### Establishment of a primary endometrial epithelial cell culturing system

**Fig 1:**
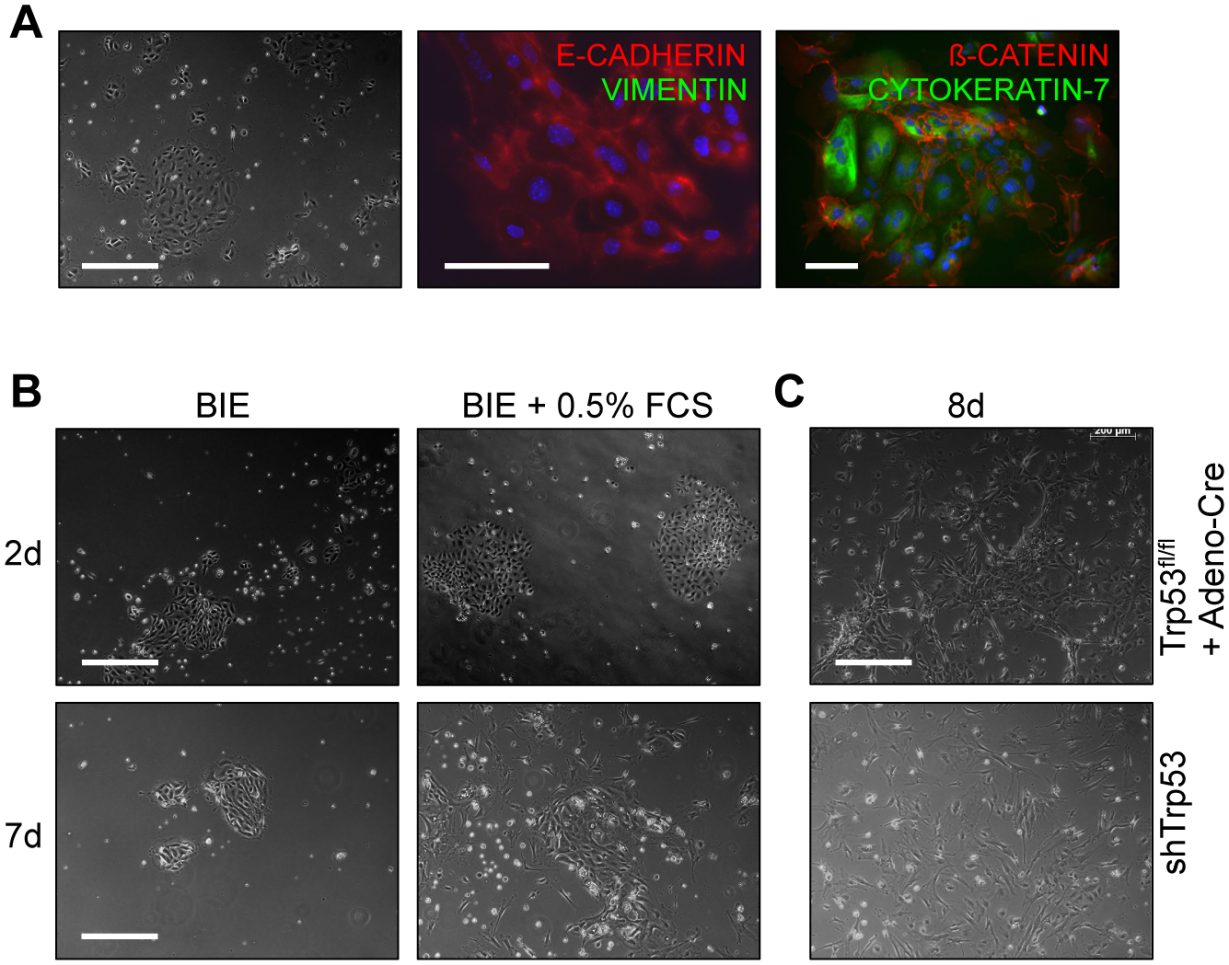
Establishment of a primary endometrial epithelial cell culturing system. (**A**) pEECs stain positively for the epithelial cell markers E-CADHERIN, ß-CATENIN and CYTOKERATIN-7 and negatively for the stromal cell marker VIMENTIN. Bright field image (10x) scale bar: 50 μm, low magnification (20x) scale bar: 100 μm and high magnification (63x) scale bar: 100 μm. (**B**) Supplementing BIE with 0.5% FCS promotes growth. BIE: basal medium supplemented with insulin, EGF, transferrin und sodium selenite. Scale bar: 50 μm. (**C**) Adeno-Cre-treated pEECs from *Trp53*^fl/fl^ mice and pEECs infected with a lentivirus expressing a miR-30-based shRNA against *Trp53* were immortalised and could be expanded but lost epithelial morphology. Scale bar: 50 μm.

To study the ability of the MuLE system to model uterine malignancies, we first focused on the most common uterine tumour type, endometrial carcinoma. We established a murine endometrial epithelial cell culture model to allow us to study endometrial tumour development *ex vivo*. Freshly isolated cultures of primary endometrial epithelial cells (pEECs) showed almost uniform postive staining for E-CADHERIN, ß-CATENIN and CYTOKERATIN-7 and, with the exception of a small number of cells, negative staining for VIMENTIN, establishing the relative purity of the epithelial isolation (Fig 1A). Next, we optimised cell culture conditions to allow the proliferation of these cells. The addition of 0.5% charcoal-stripped FCS to the medium allowed expansion of the cells into larger clusters in which cells maintained their epithelial cobblestone morphology. If FCS was not added the cells maintained their epithelial morphology but did not expand and passaging them was not possible (Fig 1B). Based on these results, we decided to culture the cells with 0.5% FCS.

The extended replicative life span which results from the inactivation of the p53 pathway is thought to represent an important event in the multistep process of endometrial carcinoma development. Given this genetic link to human endometrial carcinogenesis and additionally since pEECs fall into senescence with ongoing cultivation on plastic dishes [28], we tried to immortalise them by using primary epithelial cultures prepared from *Trp53*^fl/fl^ uteri. *Trp53* was deleted *ex vivo* by treating the cells with a Cre-expressing adenovirus. Additionally, in a parallel approach, we infected pEECs from wildtype mice with a MuLE vector expressing a miR-30-based shRNA against *Trp53* together with drug resistance. Genetic deletion or knockdown of *Trp53* allowed the cells to be expanded and passaged, but passaging still caused a change in cellular morphology to a more fibroblast-like appearance (Fig 1C), meaning that these cultures do not represent a system that can be used for long-term studies of endometrial epithelial cells.

**Fig 2:**
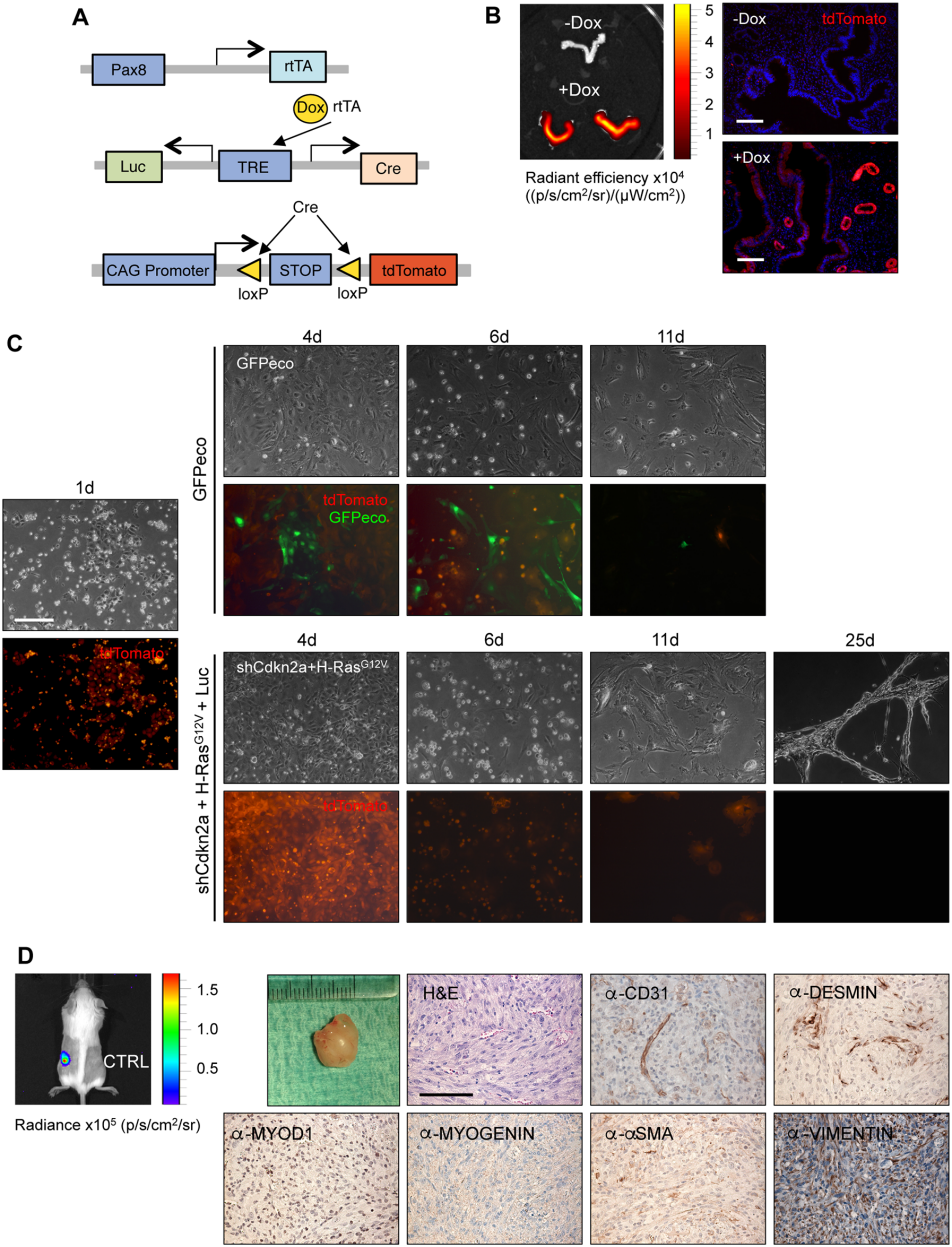
Lentiviral transduction of cultured uterine cells strongly favors stromal cells. (**A**) Schematic diagram of Pax8-rtTA-mediated doxycycline-controlled Cre-expression in Pax8-rtTA; LC1; ROSA26-lox-stop-lox tdTomato mice. (**B**) Doxycycline activates Cre-mediated recombination in glandular und luminar epithelial cells in the endometrium shown by whole organ imaging (left panel) and imaging of frozen histological sections (right panels). Scale bar: 100 μm. (**C**) Bright field and fluorescence images 1, 4, 6, 11 and 25 days after the isolation of pEECs from the uteri of doxycycline-treated Pax8-rtTA; LC1; ROSA26-lox-stop-lox tdTomato mice. pEECs were infected on day 1 with ecotropic lentiviruses expressing either GFP or sh*Cdkn2a* plus *Hras*^*G12V*^ and Luciferase. Scale bar: 50 μm. (**D**) Ability of sh*Cdkn2a* and *Hras*^*G12V*^-transduced cells to form tumours within 47 days in allograft experiments (visualised using luciferase imaging in the left panel). Right panels show H&E and immunohistochemical stainings using the indicated antibodies. Low magnification scale bar: 1 cm and high magnification scale bar: 100 μm.

We next sought to determine whether primary endometrial epithelial cells could be readily infected by MuLE viruses. For these experiments we isolated endometrial epithelial cells from uteri of Pax8-rtTA; LC1; ROSA26-lox-stop-lox tdTomato mice [25,29]. PAX8 is a transcription factor which is essential for the development of the female genital tract, including luminal and glandular endometrial epithelial cells [30]. In this mouse model, the reverse tetracycline-controlled transactivator (*rtTA*) is expressed under the control of the Pax8 promotor (*Pax8-rtTA*). In the presence of doxycycline, *rtTA* can bind to a tetracycline response element (*TRE*) which leads to the expression of Luciferase and Cre, specifically in endometrial epithelial cells, which induces the expression of tdTomato (Fig 2A). Following the administration of female mice with doxycycline to activate Cre-mediated recombination, uteri were harvested and Pax8-driven tdTomato expression in glandular and luminar epithelial cells was confirmed by *in vivo* imaging (Fig 2B) and microscopy. We isolated pEECs from the uteri of these mice and infected them with ecotropic MuLE lentiviruses expressing GFP to label infected cells, After 48 hours only a few Pax8-tdTomato-expressing cells were GFP-positive but the majority of GFP-expressing cells were Pax8-tdTomato-negative (Fig 2C, top half), demonstrating that infection of epithelial cells by MuLE viruses is rare.

We nonetheless reasoned that it might be possible to transform epithelial cells and potentially create an *ex vivo* engineered endometrial carcinoma model by infecting pEECs with an ecotropic MuLE virus expressing sh*Cdkn2a* plus *Hras*^*G12V*^, taking advantage of the fact that largely mutually exclusive genetic alterations in *KRAS*, *HRAS*, *NRAS*, *BRAF* and *NF1* occur in about 32% of human endometrial carcinomas (www.cbioportal.org) and loss of function of *Cdkn2a* in mouse cells allows escape from RAS-induced senescence and transformation [31,32]. Infection of the above-described genetically labelled pEEC cultures with the sh*Cdkn2a* and *Hras*^*G12V*^-expressing lentivirus led to the emergence of a population of apparently transformed cells that proliferated rapidly, could be sequentially passaged and formed stream-like structures in culture, however these cells were negative for tdTomato expression (Fig 2C, lower half), indicating that they are not epithelial in origin. It is known that endometrial epithelial cell cultures can be contaminated by small numbers of endometrial stromal cells and indeed, when these transformed cells were injected subcutaneously into SCID/beige mice they formed tumours with a sarcoma-like morphology. Histologically, the tumours stained negatively for CD31, DESMIN, MYOD1, MYOGENIN, alpha-SMA and positively for VIMENTIN (Fig 2D). This molecular phenotype is consistent with a diagnosis of undifferentiated sarcoma of likely mesenchymal cell origin. We conclude that while endometrial epithelial cells can be infected by MuLE viruses, albeit at low efficiency, transformation of other stromal cell types present in these cultures occurs efficiently by *Hras*^*G12V*^ overexpression and *Cdkn2a* knockdown.

### Stromal cells are preferentially transduced by lentiviruses following *in vivo* injection

**Fig 3:**
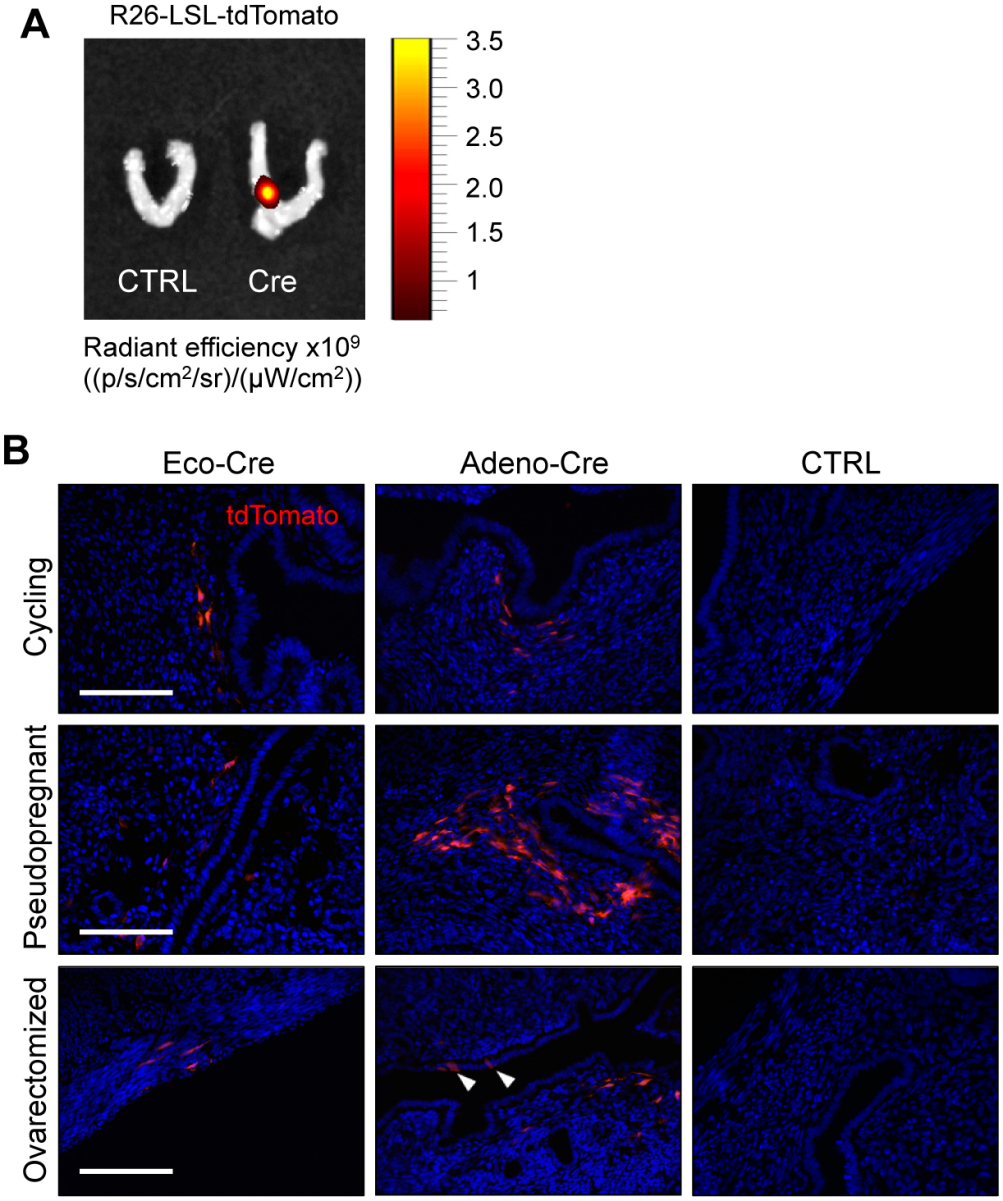
Stromal cells are preferentially transduced by lentiviruses following *in vivo* injection. (**A**) tdTomato fluorescence signal in the uterus following intrauterine injection of ecotropic virus expressing Cre. (**B**) Intrauterine injection of ROSA26-lox-STOP-lox-tdTomato mice with Cre-expressing lenti- and adenoviruses. Arrowheads indicate infected epithelial cells. Scale bar: 100 μm. CTRL indicates non-injected animals.

To study the efficacy of ecotropic lentiviral infection *in vivo*, we first injected mice with GFP-, tdTomato- or Luciferase-expressing ecotropic lentiviruses, but we were unable to detect the expression of any of these markers by immunofluorescence staining. However, using R26-lox-STOP-lox-tdTomato mice [29] as a reporter system (Fig 3A) to test cellular infection, we discovered that the injection of ecotropic lentiviruses expressing Cre into the uterus of cycling (n = 3), pseudopregnant (n = 1) and ovarectomized (n = 1) mice induced tdTomato expression in cells in the uterine stroma but did not lead to infection of endometrial epithelial cells (Fig 3B, first column). Since it has been shown that adenoviruses can infect stromal and epithelial cells *in vivo* [6,22,33], we injected adenovirus expressing Cre into the uterus of cycling (n = 2), pseudopregnant (n = 1) and ovarectomized (n = 1) mice as a positive control (Fig 3B, second column). In ovarectomized mice we were indeed able to detect tdTomato-positive epithelial cells but again there were many more positive stromal cells. We conclude that lentiviral and adenoviral intra-uterine injections strongly favour infection of stromal cells rather than epithelial cells, even in mice in which endometrial cycling was hormonally blocked (ovarectomy, pseudopregnancy) to prevent the loss of potentially infected epithelial cells.

### Intrauterine injection of MuLE viruses expressing sh*Cdkn2a* plus *Hras*^*G12V*^ causes sarcoma development

**Fig 4:**
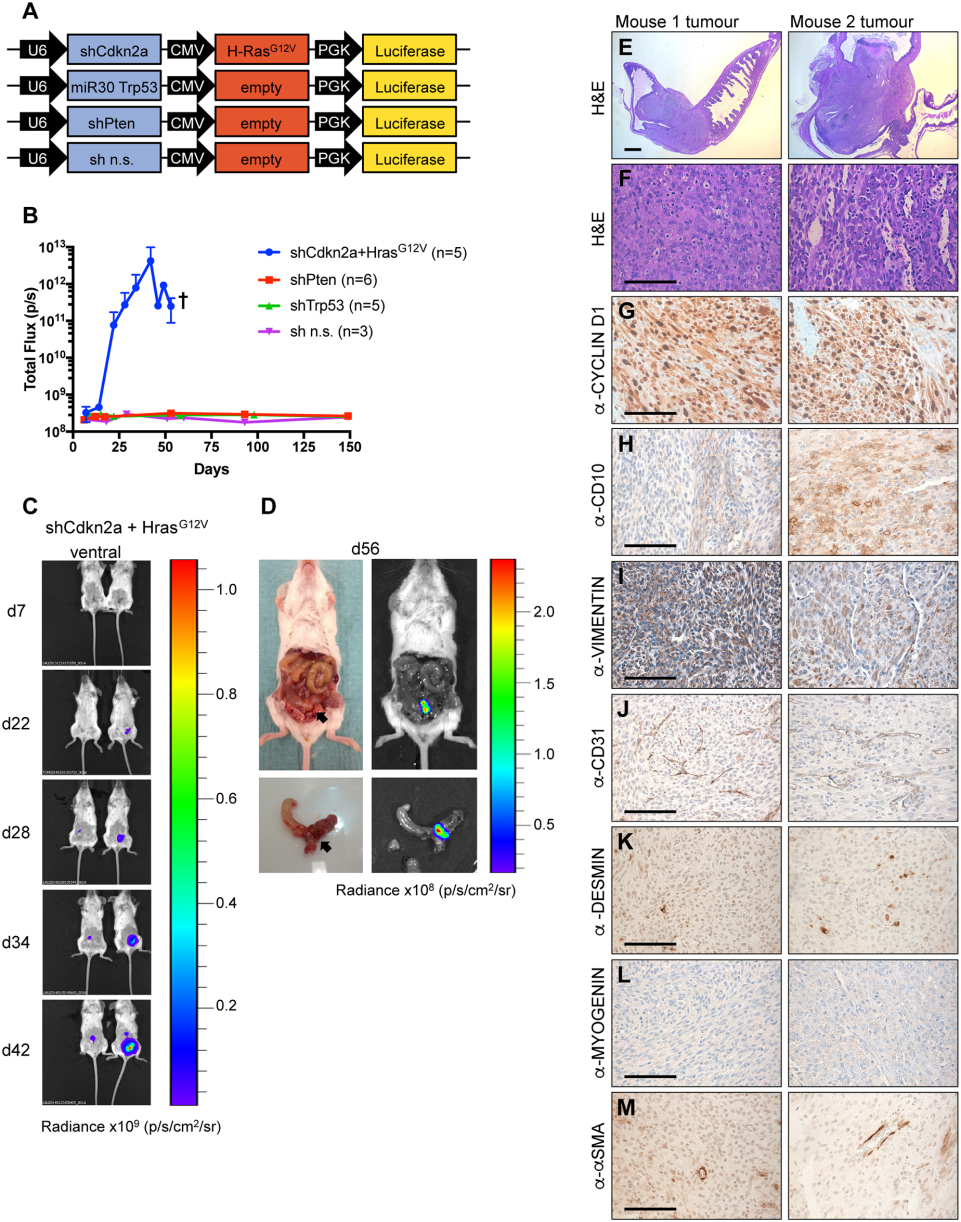
*Hras*^*G12V*^ expression plus knockdown of *Cdkn2a* causes high-grade endometrial stromal sarcomas. (**A**) Schematic of MuLE vectors simultaneously expressing a combination of shRNA against *Cdkn2a* plus expression of *Hras*^*G12V*^ or expressing shRNAs against *Trp53* or *Pten* or a non-silencing (n.s.) control shRNA. All vectors also expressed Luciferase. Numbers of mice that were injected with each vector are shown in the figure. (**B**) Quantification (mean ± SD) of luciferase signal over time. † Sacrifice of all mice in this group by this time point. (**C**) Bioluminescence imaging in two mice 7, 22, 28, 34 and 42 days after the injection of MuLE lentiviruses expressing shRNA against *Cdkn2a* together with *Hras*^*G12V*^ into the uterus of 6-8-week-old SCID/beige mice. Injected mice developed tumours (n = 4) with 80% penetrance. Median overall survival was 49 days. (**D**) Bioluminescence imaging and photographs of tumour-bearing uterus at the time of sacrifice (day 56 after injection). (**E**-**M**) H&E and immunohistochemical stainings of tumours from two different mice using the indicated antibodies. Low magnification scale bar: 1 mm and high magnification scale bar: 100 μm.

Given the data obtained above, we reasoned that it might be possible to generate a mouse model of uterine sarcomas. Since MuLE lentiviruses can be used to model undifferentiated pleomorphic sarcomas *in vivo* [24], a tumour of mesenchymal origin [22], concentrated ecotropic MuLE lentiviruses which cause either *Cdkn2a* silencing together with activation of *Hras*^*G12V*^ or *Pten* or *Trp53* silencing alone, were injected into the uterus of 4-week-old SCID/beige mice (Fig 4A). Additionally, the MuLE vectors carried a coding sequence for firefly luciferase in order to label infected cells and to trace tumour development over time. The injection of the sh*Cdkn2a* plus *Hras*^*G12V*^ expressing ecotropic MuLE lentivirus into the uterus of five SCID/beige mice caused the development of high-grade endometrial stromal sarcomas with 80% (n = 4 tumours) penetrance after less than two months (median overall survival 49 days) (Fig 4B, 4D and 4E). This process was monitored by an increase in luciferase expression over time (Fig 4C). Tumours had maximum diameters between 3–6 mm at the time of sacrifice of the mice. Tumours expressed high levels of H-RAS (S1 Fig) compared to normal uterine tissue, confirming the functionality of the MuLE vector. Histologically, the tumours were highly cellular showing a diffuse growth pattern, delicate slit-like capillary network (Fig 4J, and extensive myometrial invasion. In addition, tumors were rapidly growing (Ki-67 proliferation fraction of 10–50%, S2A Fig) with high-grade cytologic atypia, resembling endometrial stromal sarcoma. Besides hemosiderin deposits (S2B Fig), they stained positively for CYCLIN D1 (Fig 4G), diffusely positively for CD10 (Fig 4H) and VIMENTIN (Fig 4I) and negatively for CD31 (Fig 4J), DESMIN (Fig 4K), MYOGENIN (Fig 4L) and alpha-SMA (Fig 4M). These cellular and molecular features are consistent with a diagnosis of high grade endometrial stromal sarcoma. Lesions showed an absence of mucin inclusions (S2C Fig), which ruled out an adenocarcinoma phenotype. Tumours did not show staining for smooth muscle cells or collagen by van Gieson‘s staining (S2D Fig) further indicating that these tumours are not leimyosarcomas. None of the other injected vectors expressing knockdown constructs for two of the most commonly altered genes in endometrial carcinoma patients, namely *Pten* or *Trp53*, were sufficient to cause any large increases in luciferase signal over time and to induce tumour formation within 6 months after injection (Fig 4B). The development of endometrial stromal sarcoma by the injection of sh*Cdkn2a* plus *Hras*^*G12V*^ expressing ecotropic lentiviruses serves as a technical proof-of-principle that this approach can be used to model uterine sarcomas.

To further investigate the potential utility of this approach for modelling other types of uterine malignancies we generated a series of five different MuLE lentiviruses designed to reproduce the genetics of uterine leiomyomas and uterine leiomyosarcomas, namely i) overexpression of *HMGA2*, ii) expression of *MED12*^*G44S*^, iii) expression of *MED12*^*G44S*^ plus shRNA against *Trp53*, iv) expression of shRNA against *Fh* or v) expression of shRNA against *Fh* plus shRNA against *Cdkn2a*. Each vector also expressed luciferase. Each of these vectors were injected into uteri of ten or eleven mice for the *HMGA2* and *MED12* experiments and three mice for the *Fh* experiments and the mice were monitored by luciferase imaging for 9–11 months. None of these animals showed any increase in luciferase signal nor any histological abnormalities, arguing that this approach is not likely to be useful for modelling uterine leiomyomas.

## Discussion

In this study we employed a mouse genetic approach using the MuLE lentiviral gene regulatory system [24] to functionally test the ability of ecotropic lentiviruses to model epithelial and mesenchymal uterine malignancies *ex vivo* and *in vivo*.

A major problem in *ex vivo* endometrial cell culture-based studies are contaminating stromal cells, which are a byproduct of the isolation. While epithelial cells lose their proliferative capacity during ongoing cultivation on plastic, stromal cells are more easily cultured long term [28]. A Rho kinase inhibitor (ROCK), in combination with fibroblast feeder cells, induces epithelial cells from many tissues to proliferate indefinitely *ex vivo*, without the need to immortalise them [34,35]. The culture of pEECs with conditional reprogramming by feeder cells and ROCK inhibitor did not improve the culture system in our hands. Despite some minor growth in culture, pEECs displayed strong degenerative changes, such as giant intra-nuclear vacuoles, and did not survive the first passage in culture. Since it was not possible to expand and passage pEECs which retain lineage commitment and normal growth, we tried to generate immortalised cell lines through *Trp53* tumour suppressor inactivation or *Hras* oncogene activation with the use of ecotropic lentiviruses. This approach did not work, possibly due to the fact that ecotropic lentiviruses seem to efficiently infect contaminating stromal cells.

We also discovered that MuLE lentiviruses efficiently infect uterine stromal cells but not endometrial epithelial cells when injected into the uterus of cycling, pseudopregnant or ovarectomized mice. Consistent with this cellular infection spectrum, we show that intra-uterine injection of ecotropic MuLE viruses expressing oncogenic *Hras*^*G12V*^ together with knockdown of *Cdkn2a* induce high-grade endometrial stromal sarcomas. This model is analagous to a similar aggressive uterine soft tissue sarcoma model that was previously generated through a Cre-loxP germline strategy. In this model, uterus-specific deletion of *Trp53* was combined with expression of a constitutively active form of *Kras* [22]. The advantage of the MuLE system is that it is not dependent on the prior generation of germline-modified, Cre-regulatable mice and is therefore better suited to higher throughput testing of the pathological effects of different combinations of genetic alterations.

A theoretical drawback of the use of lentiviral vectors is the possibility of insertional mutagenesis. We refer to our previous detailed discussion of this issue [24] with respect to the fact that lentiviral vectors appear to be much less problematic than retroviral vectors. Data presented in this study also support the fact that insertional mutagenesis is unlikely to be a complicating factor. While MuLE viruses expressing *Hras*^*G12V*^ plus sh*Cdkn2a* induced tumours at high frequency (4 of 5 mice), we injected a total of 53 mice with numerous vectors designed to manipulate a variety of known cancer-relevant genes and did not observe a single tumour in any mouse. This underlines the specificity of the *Hras*^*G12V*^ plus sh*Cdkn2a* tumour phenotype and further demonstrates lack of non-specific tumour formation of the MuLE system in general.

Since we have previously shown that ecotropic MuLE vectors can infect a wide variety of epithelial and non-epithelial cell types [24], it remains unclear why endometrial epithelial cells are resistant to infection. It has been shown that mucin, a glycoconjugate, inhibits the entry of lenti- and adenoviruses into epithelial cells and that viral transduction could be improved by pretreating either the cells or the virus with the glycolyse hydrolase neuraminidase before infection [36,37]. It would be worth testing in the future if the pretreatment of either ecotropic lentiviruses or pEECs with neuraminidase increases epithelial cell transduction. *In vivo* studies have shown that adenoviral vector infection efficiency is influenced by the stage of the estrous cycle at the time of injection, with best results occuring during late metestrous and diestrous stages of the estrous cycle [33]. During the estrous cycle apoptosis is increased ten-fold in the luminar epithelium compared to other cell compartments [38]. Even if epithelial cells were initially infected, the high rates of apoptosis may lead to the rapid loss of these cells during the cycle. By the use of R26-lox-STOP-lox-tdTomato mice [29] as a reporter system to test cellular infection, we discovered that the injection of ecotropic lentiviruses expressing Cre into the uterus of cycling, pseudopregnant and ovarectomized mice induced tdTomato expression in stromal cells but not epithelial cells regardless of stage of the estrous cycle and the viral dose. In rodent cells a cationic amino acid transporter, termed CAT1, serves as the receptor for the envelope glycoprotein gp70 of ecotropic MMLV [39]. In the endometrium CAT1 is expressed on the basal surface of the epithelial cells [40]. Limited accessibility of CAT1 is another possible reason why we were not able to infect epithelial cells *in vivo*. Additionally, we injected Cre-liposome complexes into the uterine lumen of pseudopregnant and ovarectomized mice but also with this approach we detected reporter gene activity only in stromal cells.

Only when we injected Cre-expressing adenoviruses in ovarectomized mice, were we able to see not only tdTomato-positive stromal cells but also very few positive epithelial cells. This confirmed the earlier observations that adenoviruses infect only few epithelial cells and many more stromal cells when injected in the uterus of lacZ reporter mice [6]. The injection of an adenovirus expressing Cre into the uterus of *Pten*^fl/fl^ mice caused *Pten* deletion in both stromal and epithelial cells, however this treatment caused endometrial carcinoma development, but none of the injected uteri exhibited stromal tumours. This indicates that the deletion of *Pten* in stromal cells does not offer an advantage to the cells and therefore is not selected for in terms of causing a tumour [6]. Our studies in which injected ecotropic MuLE lentiviruses expressing sh*Pten* failed to induce stromal tumours when injected into the uterine lumen of SCID/beige mice are consistent with these data.

In summary, we discovered that ecotropic MuLE lentiviruses are able to inefficiently transduce epithelial cells *ex vivo* and that infection strongly favors stromal cells. The injection of ecotropic lentiviruses into the uterus of mice infected stromal cells but not epithelial cells regardless of stage of the estrous cycle. The MuLE system appears to be suited for use in modelling endometrial sarcomas but not carcinomas and in general represents a rapid method for performing complex genetic manipulations in uterine stromal cells *in vivo*.

## Supporting information

S1 Fig H-RAS expression in tumours. Immunohistochemical staining of two tumours from two different mice as well as adjacent normal uterine tissue using anti-H-RAS antibody.
(TIF)

S2 Fig Generation of high-grade endometrial stromal sarcomas. (**A**) Uterine tumours from two different mice show increased proliferation (Ki67 immunohistochemistry), (**B**) hemosiderin deposition (Prussian blue staining), (**C**) absence of mucin inclusions (Alcian Blue PAS staining) and (**D**) absence of strong staining for smooth muscle cells in yellow and collagen in red (van Gieson‘s staining). Scale bar: 100 μm.
(TIF)
