# Supplementary figures and images for: Oncogenic HrasG12V expression plus knockdown of Cdkn2a using ecotropic lentiviral vectors induces high-grade endometrial stromal sarcoma
Source: PLoS One. 2017 Oct 5;12(10):e0186102. doi: 10.1371/journal.pone.0186102 (PMC5628932; doi:10.1371/journal.pone.0186102)

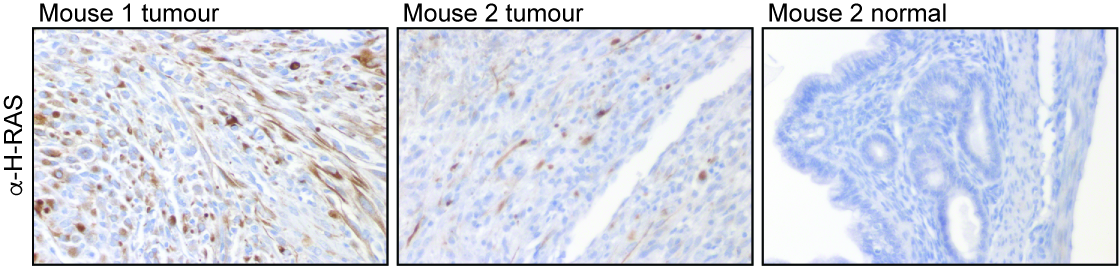

Supplement: S1 Fig — Immunohistochemical staining of two tumours from two different mice as well as adjacent normal uterine tissue using anti-H-RAS antibody. (TIF) [file pone.0186102.s001.tif]

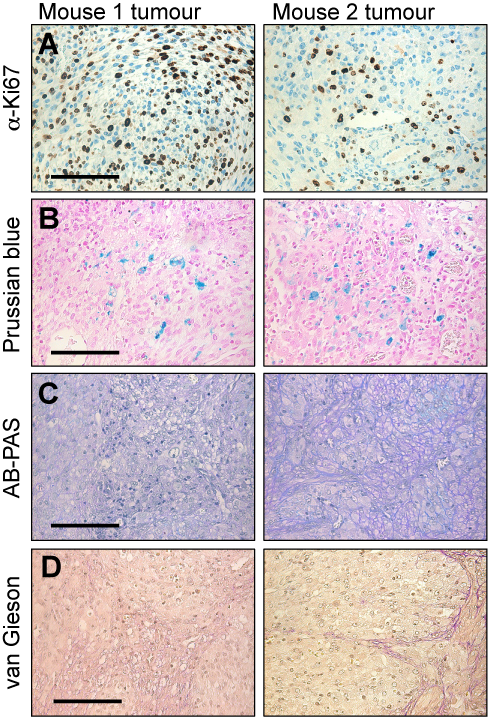

Supplement: S2 Fig — (A) Uterine tumours from two different mice show increased proliferation (Ki67 immunohistochemistry), (B) hemosiderin deposition (Prussian blue staining), (C) absence of mucin inclusions (Alcian Blue PAS staining) and (D) absence of strong staining for smooth muscle cells in yellow and collagen in red (van Gieson‘s staining). Scale bar: 100 μm. (TIF) [file pone.0186102.s002.tif]
